# A prospective evaluation of indications for neurological consultation in the emergency department

**DOI:** 10.1186/s12245-015-0074-3

**Published:** 2015-07-25

**Authors:** Christopher K Hansen, Jonathan Fisher, Nina R Joyce, Jonathan A Edlow

**Affiliations:** Department of Emergency Medicine, Icahn School of Medicine at Mount Sinai, New York, NY USA; Department of Emergency Medicine, Beth Israel Deaconess Medical Center, Harvard Medical School, Boston, MA USA; Department of Epidemiology, Brown University School of Public Health, Providence, RI USA

**Keywords:** Neurological emergency, Consultation, Discordant diagnosis, Stroke

## Abstract

**Background:**

Recognizing the diverse presentation of neurological conditions that emergency physicians encounter can be challenging, and management of these patients often requires consultation with a neurologist. Accurate diagnosis is critical in neurological emergencies because patient outcomes are often dependent on timely treatment. Our primary objective was to ascertain whether consultant neurologists understood the reason for consultation in the emergency department.

**Methods:**

The authors conducted a prospective study of a non-consecutive sample of 94 patients seen in an academic tertiary care emergency department (ED) who underwent consultation by neurologist over 4 consecutive months. At the time a consult was requested, we independently surveyed the treating ED physician for their differential diagnosis. Neurologists were also queried as to whether there was a clear indication for consultation. We then followed the patients to determine their final diagnosis and outcome.

**Results:**

The median age was 57 years (interquartile range 45–78). 45.7 % were male. The clinical reasons for all the consults were 61 % focal symptom, 12 % concern about a specific diagnosis, 9 % radiological finding, 9 % diagnostic ambiguity, and 11 % other. There was no significant difference in the rate of a final neurological diagnosis based on the clinical reason for consult (*p* = 0.13). In the 17 % of patients for whom the treating neurologist reported a lack of a clear indication for the consultation, 25 % were later admitted to a neurological service, and 69 % ultimately had a neurological diagnosis.

**Conclusions:**

Although patients with neurological emergencies can have diverse presentations, emergency physicians appear to utilize neurologic consultation appropriately. Additionally, nearly 70 % of patients for whom the consultant did not precisely understand the need for the consultation had neurological diagnoses. Time and resource constraints in the ED create challenges in making correct diagnosis.

## Background

The spectrum of neurologic emergencies that emergency physicians encounter is wide and includes complaints such as focal weakness, headache, dizziness, and seizure [1]. Diagnosing these patients can be challenging, and many of these patients require consultation with a neurologist. In addition, emergency department (ED) management of patients with neurological emergencies, including stroke, traumatic brain injury, and subarachnoid hemorrhage, continues to evolve at a rapid pace. Accurate diagnosis is critical in these patients because many treatments are time dependent [2]. Incorrect or delayed diagnosis can result in poor clinical outcomes and reduce efficiency of the health care system.

Our study had two main objectives. First, we sought to ascertain whether consultant neurologists understood the reason for consultation in the ED. Secondly, we wanted to identify the diagnostic accuracy of those cases that had neurologic consultation during care in the ED. To our knowledge, no study has systematically addressed the issues of whether neurologists are more accurate than emergency physicians in neurological diagnosis at an early point in time of the patient’s care while still in the ED.

## Methods

This prospective study was conducted in an academic tertiary care hospital emergency department with an annual volume of 52,750 over the course of 4 consecutive months. In real time, we enrolled a convenience sample of ED patients for whom neurology consultation had been ordered. Research assistants (RAs) enrolled patients for approximately 40 h per week, with one-third daytime hours, one-third evening hours, and one-third overnight hours, including weekends. The RAs carried a pager that was activated every time the neurology consultant was paged and also screened the real-time dashboard for neurology consultation requests from the ED.

The patient’s age, gender, race, chief complaint, time and date they entered the ED, and time and date they left the ED were extracted from the electronic medical record. Cases were classified as (1) initial ED visit (if this was their first ED encounter), (2) initial ED visit following outpatient referral (if they had been referred by an outpatient provider prior to arrival), or (3) transfer (if they were transferred form an outside hospital ED). If the patient had been transferred from another ED, the reason for the transfer was also recorded.

The attending ED physician, or if unavailable, the senior resident physician in charge of the patient’s care, was approached by a trained research assistant (CKH or NRJ) after the decision to place a consult had been made but prior to discussing the case with the neurologist. The ED physician was asked to classify the reason for their consultation as a focal finding or symptom, concern about a specific diagnosis, a radiologic finding, or diagnostic uncertainty. Only one reason could be selected. In addition, they were queried for the most likely diagnosis on their differential. Data was collected on a standardized data form. The ED physician and research assistant had no knowledge of the consulting neurologist’s findings at the time of query. It was not feasible to record electronic and telephonic conversations between the ED resident and neurology consultant.

Neurologic consultation occurred within 2 hours of the request and was typically placed by the junior ED resident. We recorded the time and date the consultant was called. In all cases, the neurology consultant was a second or third year resident, who reviewed the case with an attending neurologist before making recommendations. The consultant was then asked to provide a diagnosis after seeing the patient but before discussing with the attending. The consultant, after seeing the patient, was also asked the question “Do you feel you understand the reason(s) for the consult?” for which a response was entered as “yes” or “no.”

Any imaging performed while in the ED (along with the time of the study) and the results as reported by the attending radiologist were recorded. We defined a positive study as an acute finding that was thought to be the cause of the patient’s symptoms. We defined advanced neuroimaging any study beyond a non-contrast computed tomography (CT) study. These included CT angiogram, CT with intravenous contrast agent, perfusion CT, and any magnetic resonance imaging (MRI) study.

We recorded the patient’s disposition: discharged from the ED, admitted, transferred to another hospital, or left the ED against medical advice. If the patient was admitted, we recorded the service to which they were admitted, their hospital length of stay, and their final discharge diagnosis. We compared the initial ED diagnosis to the consultant neurologist’s diagnosis. Alternative neurologic diagnoses and non-neurologic diagnoses were considered discordant. Additionally, we compared the ED and consultant’s neurologist’s diagnosis to the discharge diagnosis, which we considered the “gold standard.” The study was approved by the hospital’s Institutional Review Board (Beth Israel Deaconess Medical Center IRB).

### Statistical analysis

A study sample of approximately 100 patients was planned based on the estimated number of patients expected to be enrolled during the study period. This would give the study a power to detect a 20 % difference with a beta of 0.8. All information collected in the study was aggregated into a dataset using Microsoft Excel. A second abstracter reviewed a subset of data to ensure accuracy. We used IBM SPSS statistics (version 20; IBM Corp., Armonk, NY) for data analysis and calculated medians with interquartile ranges and proportions with confidence intervals; comparisons were made with fisher’s exact tests and Mann-Whitney *U* tests.

## Results

### Patient characteristics

A total of 100 patients evaluated by a consulting neurologist in the ED were enrolled in the study. Six patients were excluded from the study because of incomplete data, leaving 94 patients for analysis. 45.7 % were male. Patients ranged in age from 20 to 94, with a median age of 57 years (interquartile range 45–78). The median length of stay in the ED for patients with neurological consultation was 6.8 h (interquartile range 5.2–8.7) as compared with the median for all ED patients of 4.7 h (interquartile range 3.5–7.9) during the study period.

Forty-two patients (44.7 %) presented directly to the ED (without being seen by another healthcare professional for their acute symptoms), 32 (34 %) were referred to the ED by another outpatient provider, and 20 (21.3 %) were transfers from another ED. Of the transferred patients, 7 (35 %) were transferred for an intracranial hemorrhage, 12 (60 %) were transferred because unavailability of a neurologist at the referring ED, and 1 (5 %) was transferred because a specific diagnosis was made at the initial facility when treatment required resources that were not available. The top chief complaints of patients in the study are listed in Table 1.

**Table 1:** Top chief complaints of study cases

	Number (%)
Focal weakness	26 (27.7)
Dizziness	15 (16.0)
Headache	14 (14.9)
Focal sensory deficit	11 (11.7)
Seizure	10 (10.6)
Visual changes	8 (8.5)
Stroke	6 (6.4)
AMS	5 (5.3)

Seventy-six (80.9 %) patients had some form of brain imaging performed and 25 (26.6 %) cases receiving some form of advanced imaging. Of the 60 patients who received a non-contrast brain CT, 15 (25 %) had a positive finding. Among the patients who received at least one form of advanced imaging, 12 (48 %) cases had a positive finding. Sixty-six (70.2 %) patients with a neurology consult were admitted with 8 (8.5 %) admitted to an intensive care unit (ICU)). This contrasts with a 38 % admission rate for all ED patients with a 5.4 % ICU admission rate (*p* < 0.001). Table 2 shows the services to which patients were admitted.

**Table 2:** Services patients where patients were admitted (n = 66)

	Number (%)
Neurology	44 (66.6)
Neurosurgery	1 (1.5)
General medicine	12 (18.2)
OB/GYN	1 (1.5)
Neurology ICU	6 (9.1)
Neurosurgery ICU	1 (1.5)
Medical ICU	1 (1.5)

### Neurological consultation

In 16 (17 %) cases, the consulting neurologist responded “no” to the question “Do you feel you understand the reason(s) for the consult?” Of these patients, 4 (25 %) were later admitted to a neurology service and 11 (68.7 %) ultimately had a neurologic diagnosis. Table 3 shows the services that patients were admitted to when the consultant neurologist did not understand the reason for the consult.

**Table 3:** Disposition when the consultant did not understand the question (n = 16)

	Number (%)	95 % CI	
Discharged	9 (56.3)	31.4	81.1
Neurology	4 (25)	3.3	46.7
General medicine	2 (12.5)	^a^	29.9
OB/GYN	1 (6.3)	^a^	18.4
Neurosurgery ICU	1 (6.3)	^a^	18.4

^a^Confidence intervals crosses 0

Clinical reasons for neurologic consultation placed by the emergency physician included 57 (60.6 %) for a focal symptom, 11 (11.7 %) for a concern about a specific diagnosis, 8 (8.5 %) for a radiological finding, and 8 (8.5 %) cases for an ambiguous presentation. There was no significant difference in the rate of neurological diagnosis based on the clinical reason for consult (*p* = 0.13), as shown in Table 4. Of the 8 patients with focal deficits but for whom the neurologist did not understand the reason for the consultation, the final diagnoses were migraine (2), orthostasis (2), stroke, dehydration, subdural hematoma, and trigeminal neuropathy.

**Table 4:** Emergency department physician clinical reason for neurological consult

	Clinical reason (%)	95 % CI		Clear indication (%)	95 % CI		Diagnostic accuracy (%)	95 % CI	
Focal symptom	57 (60.6)	50.6	70.7	49 (86.0)	76.8	95.2	47 (82.5)	72.4	92.5
Concern for diagnosis	11 (11.7)	5.1	18.3	10 (90.9)	73.6	99.9	10 (90.9)	73.6	99.9
Radiologic finding	8 (8.5)	2.8	14.3	8 (100.0)	^a^	^a^	8 (100.0)	^a^	^a^
Diagnostic ambiguity	8 (8.5)	2.8	14.3	4 (50.0)	14.6	85.4	7 (87.5)	64.1	110.9
Other reasons	10 (10.6)	4.3	17.0	7 (70.0)	41.0	99.0	8 (80.0)	54.7	105.3

Clear indication—number of cases in which the consultant neurologist understood the reason for consult

Diagnostic accuracy—number of cases with concordant diagnosis between the ED physician and the consultant neurologist

^a^Confidence interval crosses 0 or 100

### Diagnostic accuracy

Of the 94 cases in our study, 80 (85.1 %) had a neurologic diagnosis that was concordant between the ED physician and the consultant neurologist. Among the 14 (14.9 %) discordant cases, 9 were given a neurologic diagnosis by the consultant that was a different than the one given by the ED physician (Table 5) and all were concordant between the consultant and the final discharge diagnosis. The remaining 5 cases had a final non-neurologic diagnosis (Table 6). Eight cases (57 %) had an initial ED diagnosis of stroke or TIA and were later given a different diagnosis, including two, which were non-neurologic in nature. Additionally, of the 14 discordant cases, 4 also had discordance between the consultant neurologist and the final diagnosis. Of these 4, the initial ED diagnosis was also not concordant with the final diagnosis. There was no statistical difference in the median length of stay in the emergency department of the discordant cases (7.3 h, interquartile range 3.4–10.0), compared to the concordant cases (6.8 h, interquartile range 5.3–8.7) (*p* = 0.89).

**Table 5:** Discordant alternative neurologic diagnoses

Case	ED diagnosis	Final diagnosis	Disposition
1	Cerebellar stroke	Gait disorder	Discharged
2	Migraine	Bell’s palsy	Discharged
3	Stroke	Bell’s palsy	Discharged
4	TIA	Seizure	Discharged
5	TIA	Trigeminal neuropathy	Discharged
6	Cerebellar stroke	Benign positional vertigo	Admitted-medicine
7	Cord compression	Non-neurologic urinary retention	Admitted-medicine
8	Pontine hemorrhage	Epilepsy, with active partial seizures	Admitted-neurology
9	TIA	Complicated migraine, autonomic instability	Admitted-neurology

**Table 6:** Discordant non-neurologic diagnoses

Case	ED diagnosis	Final diagnosis	Disposition
1	Stroke	Dehydration	Discharged
2	Peripheral vertigo	Orthostatic hypotension	Discharged
3	Gait abnormalities	Non-neurological gait disorder	Admitted-medicine
4	Stroke	Altered mental status due to medication	Admitted-neurology
5	Neuropathy	Post-surgical abdominal pain	Admitted-OB/GYN

## Discussion

Because neurological complaints represent significant proportion of ED visits, emergency physicians must be capable of accurately assessing and managing them. The top neurologic chief complaints in our study (focal weakness, dizziness, and headache) are similar to several other studies, both in the US and internationally [1, 3, 4]. The vast majority of cases in our study (85.1 %) had a neurologic diagnosis that was concordant between the ED physician and the final diagnosis, which reflects the ability of emergency physicians to assess and diagnoses neurologic complaints.

The overall rate of correct diagnosis stroke and TIA combined in our study was 64 %. While this number is lower than previous studies have reported [5–7], it should be noted that by protocol, our threshold for a neurological consultation is extremely low for patients presenting within 9 h of symptom onset, a factor that tends to decrease diagnostic accuracy. Table 5 reveals six cases that were initially believed to be stroke/TIA by the emergency physician but later received final diagnoses of Bell’s palsy, seizure, trigeminal neuralgia, benign positional vertigo, gait disorder, or migraine. Furthermore, two cases that were initially thought to be stroke had the non-neurologic diagnoses of dehydration and altered mental status due to hydromorphone as shown in Table 6. In these cases, the error in diagnosis was mistaking a serious condition for a more benign one. However, this type of error favors increased patient safety. Two cases, one of Bell’s palsy and one of benign positional vertigo, were misdiagnosed as cerebellar stroke, a potentially difficult diagnosis [8]. These two patients were older (mean age of 74.5) and had significant vascular risk factors.

Previous international studies have examined overall diagnostic accuracy within their particular models of emergency care. A French study reported 37.3 % false positive rate and a 36.6 % false negative rate in neurological diagnosis in the ED [3]. A similar study reported 35.7 % discordance in neurological diagnoses in a Canadian emergency department [9]. However, these studies have serious methodological flaws, such as selection bias, diagnostic inclusion bias, and observer bias. Even studies that apply fairly rigorous research methods are severely limited by the fact that they analyzed misdiagnosis retrospectively (among patients diagnosed with a particular condition) rather than prospectively (among patients presenting with a particular symptom).

Studies of diagnostic accuracy of stroke by emergency physicians show mixed results. It has been shown that academic and community ED physicians are able to identify stroke and TIA patients with greater than 90 % accuracy [5, 7]. Additionally, emergency physicians in this study were able to identify 100 % of patients with hemorrhagic stroke on radiographic findings [5]. A prospective Portuguese study similarly found that emergency physicians at an academic medical center had 91 % accuracy in identifying stroke, despite the fact that only 87 % of the patients had a non-contrast head computed tomography (CT) scan [6]. Other studies have reported a higher misdiagnosis rate of stroke by emergency physicians. Prabhakaran et al. reported a 60 % prevalence in the misdiagnosis of TIA [10]. A more recent retrospective cohort study performed at a single academic center in Cleveland, Ohio, reported that 36 % of patients had a discordant diagnosis of TIA between emergency physicians and neurologists [11].

Some authors have suggested shortcomings in the evaluation of neurological symptoms by emergency physicians. Caplan [12] opined that emergency physicians are deficient in diagnostic reasoning and neurological exam skills and do not understand the limitations of CT scans with respect to neurological conditions. Similarly, Manno [13] reported that emergency physicians encounter difficulty, particularly with diagnosis and management of ischemic stroke, subarachnoid hemorrhage, and status epilepticus. It is important to consider, however, that neurologists and emergency physicians practice in very different environments under very different constraints [14]. Limited time and frequent distractions are just two of the everyday obstacles to diagnostic accuracy in the ED. Consultants are often able to spend considerably more uninterrupted time examining a patient where they apply the significant expertise gained from their specialty training. They have access to incremental history and diagnostic test results, which often evolves even over the hours of the ED visit and which were not available to the emergency physician at the time of their initial diagnosis.

Because many neurological syndromes evolve over time, the practical realities of emergency medicine may overestimate misdiagnosis rates. For example, a patient presenting with a visual field defect may initially appear to be having a stroke at the time of consultation. However, by the time the neurologist’s assessment is complete, the patient may have developed a headache, helping to confirm the diagnosis of migraine. Similarly, a patient may arrive with a prehospital emergency medical services history of an abrupt onset of weakness an hour ago, but later history from family may indicate a gradual onset over days. Emergency physicians are taught to employ the “dangerous condition first” approach to diagnosis in which alternative diagnoses of lesser severity are considered only after life or limb threatening conditions are ruled out. This may contribute to error in diagnosis by mistaking a serious condition for a more benign one, but this type of error always favors increased patient safety.

We found a low rate of misdiagnosis of neurological emergencies. Diagnosis of ischemic stroke, however, appears to remain a challenge for emergency physicians. Interestingly, a survey of emergency medicine residency programs reported only 35 % require a neurology or neurosurgery rotation [15]. Future studies should be focused around the language of consultation. Do providers requesting consults frame the question in as specific way as possible? If the consult is requested for dizziness, but the requestor neglects specific details to focus the consultant, does this affect patient care and diagnosis? Importantly, education in emergency medicine residency programs must ensure thorough coverage of the approach to the diagnosis and initial management of ischemic and hemorrhagic stroke syndromes.

### Limitations

There are a number of potential limitations in our study. This study was conducted at a single academic tertiary care hospital, so our results may not be generalizable to other locations, including community emergency departments where access to neurologic consultation may be very different. Additionally, our study did not examine neurosurgical consultation, which frequently manages conditions such as subarachnoid hemorrhage and traumatic brain injury in the emergency department.

A small number of patients included in our study were transferred from another emergency department and may have had imaging and workups completed at the outside facility, which patients on first presentation to our ED did not have, a potential source of bias in our study. Additionally, since neurologic consultation may have occurred while the patient was simultaneously being worked up, the neurologist may have already known the results of laboratory test or imaging when giving their diagnosis, as well as know what diagnosis the ED physician was suspicious of, while the ED physician was only able to base his diagnosis on seeing the patient fresh. This is a possible source of observer bias for the neurologist and a possible confounder to diagnostic accuracy. Because our study was a convenience sample, there is also the potential for selection bias and inclusion bias.

While we chose to use the collective decision of the ED treatment team, which often includes an intern, senior resident, and attending, we did not examine the specific level of experience of each provider. We were also not able to record the exact language of the consult that was transmitted by telephone between the ED resident and the neurology consultant, and thus, the potential exists that subtleties of the decision of the ED team to place the consult may have been left out. Additionally the question “Do you feel you understand the reason for the consult?” may have allowed for aberrant interpretations by the consulting neurologist.

Although our data shows we did not miss any strokes, we had a small sample size. Moreover, our analysis did not find any stroke misdiagnosis. Our results are similar to that which has been clearly documented in the literature; however, we cannot and do not conclude that stroke diagnosis is perfect.

## Conclusions

Although patients with neurological emergences can have diverse presentations, emergency physicians appear to utilize neurologic consultation appropriately. Emergency medicine training in neurology should place special emphasis on the evaluation of patients with weakness, seizures, headache, and dizziness and emphasize acute ischemic stroke presentations.
